# Recent advances in polyhydroxyalkanoate production from volatile fatty acids derived from food waste fermentation

**DOI:** 10.3389/fmicb.2025.1693596

**Published:** 2025-10-17

**Authors:** Xue Tao, Shiqi Liu, Longyi Lv, Li Sun, Guangming Zhang, Jinsong Liang, Wenxiu Zou

**Affiliations:** ^1^School of Resources and Environmental Engineering, Moutai Institute, Renhuai, Guizhou, China; ^2^School of Energy & Environmental Engineering, Hebei University of Technology, Tianjin, China; ^3^Northeast Institute of Geography and Agroecology, Chinese Academy of Sciences, Harbin, China

**Keywords:** bioplastics, polyhydroxyalkanoates, food waste, waste valorization, mixed microbial communities

## Abstract

Polyhydroxyalkanoates (PHAs) are promising green substitutes for traditional plastics, offering good biodegradability and biocompatibility. PHA production using volatile fatty acids (VFAs) obtained from food waste fermentation not only provides a new way to utilize food waste resources but also reduces the PHA production cost. However, a review of mechanisms, technical processes, key influencing factors, and techno-economic analysis of food waste-VFAs-PHA production is lacking. Thus, this review elucidates the microorganisms that synthesize PHA and their associated metabolic pathways. A technical process of food waste-VFAs-PHA generation was proposed. Research status in this field was summarized. Meanwhile, the influencing factors of PHA synthesis based on VFAs were discussed. Additionally, techno-economic and environmental analyses of the food waste-VFAs-PHA process were covered. Finally, the challenges and prospects of future work were proposed. This review provides new ideas and theoretical guidance for achieving industrial production of low-cost PHA and the value-added transformation of food waste.

## 1 Introduction

With the increase in white pollution and depletion of petroleum resources, it is imperative to produce excellent and sustainable plastic to replace petrochemical plastics (Chouhan and Tiwari, 2025). Polyhydroxyalkanoates (PHA) are intracellular polyesters synthesized by microorganisms (Xiong et al., 2023; Zhang X. et al., 2025). With their renewable, biodegradable, and biocompatible properties, PHAs are good alternatives to traditional plastics, offering versatile applications in the fields of biomedicine, food packaging, agriculture, aquaculture, and tissue engineering (Liu J. et al., 2024; Zhou et al., 2023). Currently, sugars (glucose, sucrose), alcohols (methanol, ethanol), organic acids (acetate, propionate, butyrate, valerate), among others, are utilized as raw materials for PHA synthesis (Lim et al., 2023; Du et al., 2025). However, due to the high cost of substrates for PHA synthesis (Goswami et al., 2023), there is an urgent need to find cheaper substrates.

Biomass waste is one of the most abundant renewable resources. Volatile fatty acids (VFAs) derived from fermented biomass waste as carbon sources for PHA synthesis not only reduce the cost but also promote waste valorization, offering both environmental and economic benefits (Zhang et al., 2024). Various inexpensive carbon sources have been used for PHA synthesis, including straw biomass, food waste, and sewage sludge (Bhavana et al., 2025; Sekoai et al., 2022). Among these biomass wastes, food waste is more suitable as a substrate for PHA precursor synthesis owing to its richer organic matter content (Zhang X. et al., 2025). (Raunhan et al. 2023) used acetate and propionate derived from the fermentation of food waste for PHA production by *Thauera mechernichensis*, and PHA content was 24.0%. Zhang G. et al. (2025) utilized food waste digestate as feedstock for photosynthetic bacteria, and the PHA content reached 40.8%. Thus, VFAs obtained from food waste to generate PHA are a promising resource utilization pathway.

Currently, the PHA generation from food waste as a substrate has been reviewed, focusing on the treatment, disposal, composition, hydrolysis, and pretreatment of food waste, as well as PHA synthesis (Chavan et al., 2023; Liu et al., 2025). However, the mechanisms, key influencing factors, technological processes, and techno-economic analysis in VFAs obtained from food waste for generating PHA are often neglected. In this review, we outlined the composition, classification, microorganisms, and synthetic pathways of PHA from food waste-based VFAs. A complete technological process for food waste–VFAs–PHA generation was presented. Meanwhile, the key factors in the PHA synthesis from food waste-based VFAs and recent advances in this field were examined. Furthermore, we evaluated the techno-economic and environmental analyses of food waste–VFAs–PHA generation. Finally, the current challenges and prospects were presented.

## 2 Composition and classification of polyhydroxyalkanoates

PHAs are a class of macromolecules stored in bacteria and archaea that provide additional carbon sources for microbial growth at nutrient-limited conditions, accumulating in the cell cytoplasm in the form of granules (Ishak et al., 2021). PHA can be divided into short-chain length PHA (SCL-PHA) and medium-chain length PHA (MCL-PHA) based on the number of carbon atoms contained in the monomer. Length of monomer carbon chain of SCL-PHA is generally 1–5, and length of monomer carbon chain of MCL-PHA is between 6 and 14. Long-chain PHA is not common owing to the complexity of synthesis, and poly-3-hydroxybutyric acid (PHB) and poly-3-hydroxyvaleric acid (PHV) are common SCL-PHA (Yim et al., 2023). In general, natural microorganisms can only accumulate either SCL-PHA or MCL-PHA. PHA can also be classified into homopolymers, random copolymers, block copolymers, and so on, according to the arrangement of monomers. Material properties of PHA are determined by monomer composition, molecular weight, and its distribution. The properties of different PHA types vary greatly, and bioplastic products made from them exhibit corresponding variations in their properties (Ilhami et al., 2025; Sabapathy et al., 2020).

## 3 Polyhydroxyalkanoate-producing microorganisms

Currently, both pure and mixed microbial cultures are used for PHA synthesis. Fast-growing, adaptable, high-yielding, and efficient PHA synthesis strains or communities are a key area of research, representing an important direction. To date, nearly 100 genera of microorganisms have been isolated from different natural ecosystems for PHA generation (Fu et al., 2023). These strains, screened from natural ecosystems, have demonstrated a good ability to produce PHA (Table 1), including *Halomonas, Cupriavidus, Pseudomonas, Methylotrophs*, and *Azoobacter*, among others. With the development of molecular biology, some genetically engineered bacteria capable of producing PHA efficiently have been constructed. For example, the recombinant *Escherichia coli* constructed by Jung et al. (2019) produced PHA accounting for up to 66% of the dry cell weight, which was 25–28 times higher than that of the wild strain. The disadvantage of pure microbial cultures is the high cost. Thus, strategies such as increasing strain yields, utilizing inexpensive materials, and developing open fermentation techniques have been employed to reduce the production cost of PHA (Chavan et al., 2023; Fu et al., 2023).

**Table 1:** PHA producing microorganisms obtained by natural screening.

**Genus level**	**Species**	**Source**	**PHA content (wt%)**	**References**
*Pseudomonas*	*Pseudomonas* sp. strain P16	Soil	84.0	Aljuraifani et al., 2019
	*Pseudomonas mendocina* PSU	Sediment	79.7	Chanasit et al., 2016
*Cupriavidus*	*Cupriavidus* sp. USMAA2-4.	Soil	68.0	Kek et al., 2010
	*Cupriavidus necator*	Soil	76.8	Morlino et al., 2023
*Halobacterium*	*Halomonas venusta* KT832796	Marine	88.1	Stanley et al., 2018
	*Halomonas bluephagenesis*	Marine	86.0	Ling et al., 2018
	*Halomonas alkaliantarctica*	Saline sediment	20.1	Mozejko-Ciesielska et al., 2023
	*Halomonas halophila*	Saline soil	74.7	Kourilova et al., 2021
*Bacillus*	*Bacillus thuringiensis* A102	Red ant intestines	87.4	Gowda and Shivakumar, 2014
	*Bacillus megaterium*	Mangrove forest	62.0	Mohapatra et al., 2020
*Methylobacterium*	*Methylobacterium* sp. 1805	Oil field	86.6	Wang et al., 2020
*Paracoccus*	*Paracoccus* sp. LL1	Sediment	72.4	Sawant et al., 2015
*Enterobacter*	*Enterobacter* sp. TS1L	Textile wastewater	81.2	Rakkan and Sangkharak, 2020
*Streptomyces*	*Streptomyces toxytricini* D2	Soil	86.7	Narayanan et al., 2021

PHA synthesis using mixed strains can reduce the risk of contamination by stray bacteria and expand the range of carbon source selection, which is an important strategy for synthesizing PHA with high efficiency and low cost (Pesante and Frison, 2023). Currently, the most common pattern of mixed strains is the combination of PHA-producing bacteria. A mixed microbial community based on symbiotic or mutualistic relationships can overcome the low PHA accumulation caused by the intermediate accumulation (Thamarai et al., 2024). Meanwhile, the mixed microbial community domesticated from activated sludge does not require the extermination of stray bacteria, offering advantages such as adaptability, simplicity, and applicability to various inexpensive carbon sources (Fu et al., 2023).

The synthesis of PHA from organic waste in a mixed microbial community is generally divided into three stages (Du et al., 2025). The first stage involves the anaerobic fermentation of organic waste to produce VFAs. The second stage involves the domestication of mixed communities from activated sludge, which have the ability to efficiently produce PHA. This is the key stage that determines the efficiency of PHA production. The third stage involves PHA production by the domesticated mixed microbial communities using VFAs-rich digestate under optimal conditions (pH, temperature, dosing batch time, and synthesis time). Wu et al. (2023) reported that PHA content reached 47.9% in a pilot plant after 180 days of operation in a three-stage process, indicating the potential for large-scale production of PHA by a mixed microbial community. However, the failure of mixed microbial culture to achieve industrial production is primarily attributed to their low PHA yield and the difficulty in maintaining the stability of long-time fermentation (Matos et al., 2021).

## 4 Biosynthesis of polyhydroxyalkanoate

### 4.1 Metabolic pathways

**Figure 1:**
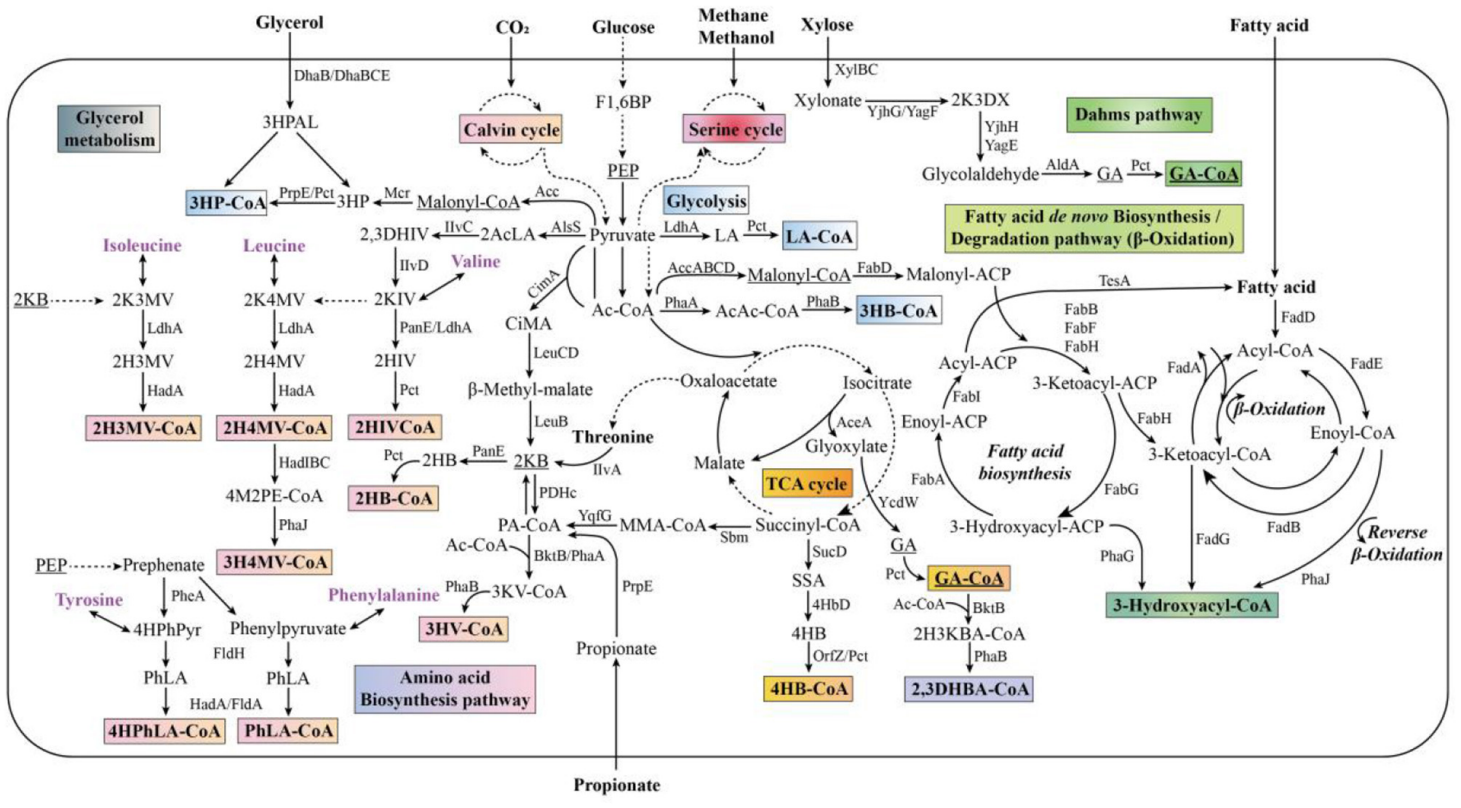
Main metabolic pathways of PHA generation (Adapted from Choi et al., 2020).

There are three main pathways for PHA biosynthesis, namely synthesis of PHA using acetyl-coenzyme A (CoA) as a precursor (pathway I), the β-oxidation pathway of fatty acids (pathway II), and the *de novo* synthesis of fatty acids (pathway III) (Figure 1). In pathway I, two acetyl-CoA molecules are first condensed by β-ketothiolase to form acetoacetyl-CoA, which is then converted to (R)-3-hydroxybutyryl-CoA by acetoacetyl-CoA reductase. Finally, (R)-3-hydroxybutyryl CoA is catalyzed by PHA synthase (PhaC) for PHB generation. Pathway I is the most common one for PHA biosynthesis, and *C. necator* is the model strain that synthesizes PHA in this manner (Nygaard et al., 2025). In pathway II, fatty acids are first activated to esteroyl CoA, which then enters the β-oxidation pathway and is converted from (S)-3-hydroxy esteroyl CoA to (R)-3-hydroxy esteroyl CoA by enoyl CoA hydratase, and then finally synthesized into PHA catalyzed by PhaC. The majority of *Pseudomonas* spp. can obtain energy to sustain cell growth via the β-oxidation pathway of fatty acids (Kim et al., 2007). In pathway III, intermediates of the fatty acid *de novo* synthesis pathway are involved in the synthesis of PHA. 3-Hydroxyesteroyl ACP-CoA transferase (PhaG) acts as a key enzyme to convert (R)-3-hydroxyesteroyl ACP to (R)-3-hydroxyesteroyl CoA, which can eventually be catalyzed by PhaC to synthesize PHA. Among these three pathways, pathway I is more compatible with simple carbon sources, whereas pathways II and III allow for the utilization of complex substrates, such as fatty acids (Choi et al., 2020). In addition to the three major pathways, other PHA synthesis pathways exist in microorganisms, as well as new pathways constructed through genetic engineering. For example, 3-hydroxyvaleryl-CoA was genetically engineered for poly(3-hydroxybutyrate-co-3-hydroxyvalerate) (P3HB-co-3HV) synthesis from oxaloacetate (Meng et al., 2014), and 4-hydroxybutyryl CoA was provided for the synthesis of poly(3-hydroxybutyrate-co-4-hydroxybutyrate) (P3HB-co-4HB) from succinyl-CoA (Li et al., 2010).

### 4.2 Process of synthesizing PHA from food waste

**Figure 2:**
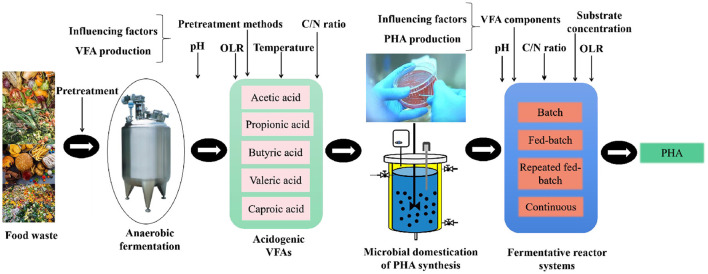
Process of food waste-VFAs-PHA generation.

Food waste contains a variety of organic compounds, including carbohydrates, proteins, lipids, and cellulose, which can be used as cheap substrates for VFA production, thereby generating PHA (Valentino et al., 2021). Generally, this process can be divided into three steps (Figure 2). (1) Hydrolysis and acidification of food waste to generate VFAs-rich digestate; (2) cultivation of efficient pure strains or enrichment of efficient mixed microbial culture for PHA synthesis; and (3) microorganisms synthesizing PHA using VFAs-rich digestate.

#### 4.2.1 Production of PHA precursors

VFAs are intermediate products in anaerobic fermentation of food waste (Chacón et al., 2024). Typically, the organic matter in food waste exists in the form of solid particles, which is not conducive to its decomposition and utilization (Moza et al., 2022). The hydrolysis efficiency of food waste becomes the limiting step (Bourgeois et al., 2025). Thus, suitable pretreatment methods can make the solid organic matter easy to decompose. The pretreatment methods include physical, chemical, biological, and combined pretreatments (Chavan et al., 2024; Kumar Biswal et al., 2020). These pretreatments can decrease the particle size of food waste and increase the reaction contact area, thus accelerating the hydrolysis process (Gallego-García et al., 2023). Meanwhile, pretreatments can provide more suitable environments for microorganisms, enhance the biochemical reaction rate, and promote the hydrolysis of starch, cellulose, and other organic matter (Sagar et al., 2024). Although physical, chemical, and biological pretreatment methods can effectively enhance the hydrolysis efficiency of food waste, their potential for industrial application varies significantly, depending on the trade-off between cost, efficiency, and environmental impact. In addition, improving some fermentation factors, such as pH, temperature, and organic load rate (OLR), is beneficial to enhancing the hydrolysis of food waste (Zhang et al., 2023a,b).

The inherent properties of food waste (such as high salinity, high oils, variability in composition, and nutrient imbalance) also influence the VFA production. Sodium chloride in food waste had an inhibitory effect on VFA production, and the inhibition significantly increased when the salt concentration exceeded 6 g/L (Huang et al., 2022; Li et al., 2023). The sodium chloride concentration of food waste as a substrate for VFA production should be less than 6 g/L. Sodium chloride not only affects the hydrolysis and acidification of food waste, but also impacts subsequent PHA production. Screening and domesticating salt-tolerant PHA-producing strains (e.g., *Halomonas*) or microbial communities is a viable approach (Muigano et al., 2025). Large amounts of oil in food waste also inhibited the acidification process, which was mainly manifested in a reduction in VFA concentration and a lag in VFA production (Salama et al., 2019). Oils were hydrolyzed into long-chain fatty acids, which attached to microbial surfaces, thereby reducing the rate of substrate translocation by microorganisms (Li et al., 2023). Noted that long-chain fatty acids were solubilized into short-chain fatty acids by microorganisms, which further increased the VFA production (Watson et al., 2021). Thus, the appropriate oils may improve the VFA generation efficiency and modulate the VFA fractions in the anaerobic fermentation of food waste.

In addition to high salt and oils, the composition variability and nutrient imbalance of food waste also affect VFA production, further affecting the production of PHA (Sagar et al., 2024). The composition of food waste fluctuates significantly due to seasonal variations, geographical differences, and variations in dietary habits. The uncertainty surrounding carbohydrate, protein, carbon-to-nitrogen (C/N) ratio, and trace element content in food waste poses substantial challenges to the stability and reproducibility of PHA production. For such situations, source separation and pretreatment of food waste are key strategies for achieving efficient homogenization (Chavan et al., 2024). Meanwhile, the mixed waste co-fermentation strategy (such as carbon-rich agricultural residues, nitrogen-rich livestock manure, and buffering sewage sludge) not only balances the C/N ratio but also provides the trace elements and growth factors (Soon et al., 2025). This supports a richer and more stable microbial community structure, thereby enhancing the resilience against substrate fluctuations of the system.

#### 4.2.2 Microbial domestication/microbial culture enrichment

Different screening and fermentation media are used to select efficient strains that can synthesize PHA using VFAs as carbon sources. Currently, some strains such as *Pseudomonas putida* and *C. necator*, have been used industrially to produce PHA using VFAs as substrates (Martín-Pascual et al., 2021; Morlino et al., 2023). Mixed-strain fermentation can reduce the risk of contamination and expand the range of carbon source selection, which is an important strategy for synthesizing PHA efficiently and cost-effectively (Du et al., 2025). Meanwhile, the symbiotic or mutualistic relationship between strains in building a mixed-strain fermentation system can relieve the excessive accumulation of intermediate products, thereby further promoting PHA accumulation (Soto et al., 2019). However, due to the complex composition of food waste digestate (including high salt, ammonia nitrogen, and sulfides), it requires extensive pretreatment and sterilization in pure microbial culture, presenting significant engineering challenges that remain far from being addressed in large-scale applications. For this food waste digestate, a mixed microbial culture is currently considered to offer greater practical feasibility and development potential.

The domestication of mixed microbial culture in activated sludge aims to control the supply of carbon sources, thereby repeatedly placing the microorganisms in a state of “feast-famine,” which enhances the ability to synthesize PHA (Fu et al., 2023). In the “famine” state, microorganisms can convert the excess carbon source into PHA stored in bacterial cells. In the “feast” state, microorganisms that have synthesized PHA can utilize PHA as a carbon source for growth and metabolism, while the growth and metabolism of microorganisms that cannot synthesize PHA are inhibited due to a lack of carbon sources, thus enriching the PHA synthesizing communities in the activated sludge (Rajesh Banu et al., 2021). Among them, control of feast-famine time in microbial domestication has been recognized as a key factor in successful domestication, with the duration of famine being less than 25% of the full domestication cycle Jia et al., 2013; Liao et al., 2018). Based on the above, the microbial quality of PHA synthesis by aerobic dynamic drainage is higher than that of other microorganisms, thus increasing the sedimentation of microorganisms and facilitating the enrichment of microbial communities for efficient PHA synthesis (Chen Z. et al., 2015).

#### 4.2.3 PHA synthesis

The addition of VFAs derived from food waste to domesticated mixed microbial culture or enriched pure culture systems achieves an efficient PHA synthesis by controlling the appropriate fermentation conditions. The PHA synthesis reactors are classified into batch, fed-batch, and continuous (Kumar et al., 2019). Batch reactors are commonly used in PHA synthesis due to their simplicity of operation and cost-effectiveness. However, the microorganisms are “famine” at the end of the fermentation due to the inability to continuously add carbon sources, resulting in the synthesized PHA being utilized as carbon sources (Sekoai et al., 2022). Fed-batch reactors can make up for the shortcomings of batch reactors by replenishing the nutrients during fermentation, reducing the accumulation of by-products, and increasing the PHA yield. However, the design and fabrication of a fed-batch reactor are complex, does not readily produce PHA on a large scale (Du et al., 2025).

Continuous reactors are much simpler in design and operation, and can also completely compensate for the disadvantage of insufficient nutrients, thereby stabilizing mixed microbial cultures during the PHA synthesis (Sekoai et al., 2022). Koller and Muhr (2014) used continuous fermentation to obtain PHA, which compensated for the unpredictable quality and low yield of the fed-batch. Bhalerao et al. (2020) produced PHA in a continuous reactor using yeast wastewater as a carbon source, and achieved 65% PHA accumulation, which was slightly lower than the batch reactor, but the biomass growth increased 4-fold and the theoretical yield reached 270 t/year. In addition, Matos et al. (2021) achieved the world's highest PHA content and productivity (80.5% and 8.1 g PHA/L·day) in a pilot plant using fruit waste as substrate in a continuous reactor. However, microbial function is prone to degradation, and substrate utilization is low during continuous fermentation. Currently, few studies have been conducted on the continuous fermentation of PHA generation using food waste digestate, which needs further study.

### 4.3 Factors affecting food waste-derived VFAs-sourced PHA production

**Figure 3:**
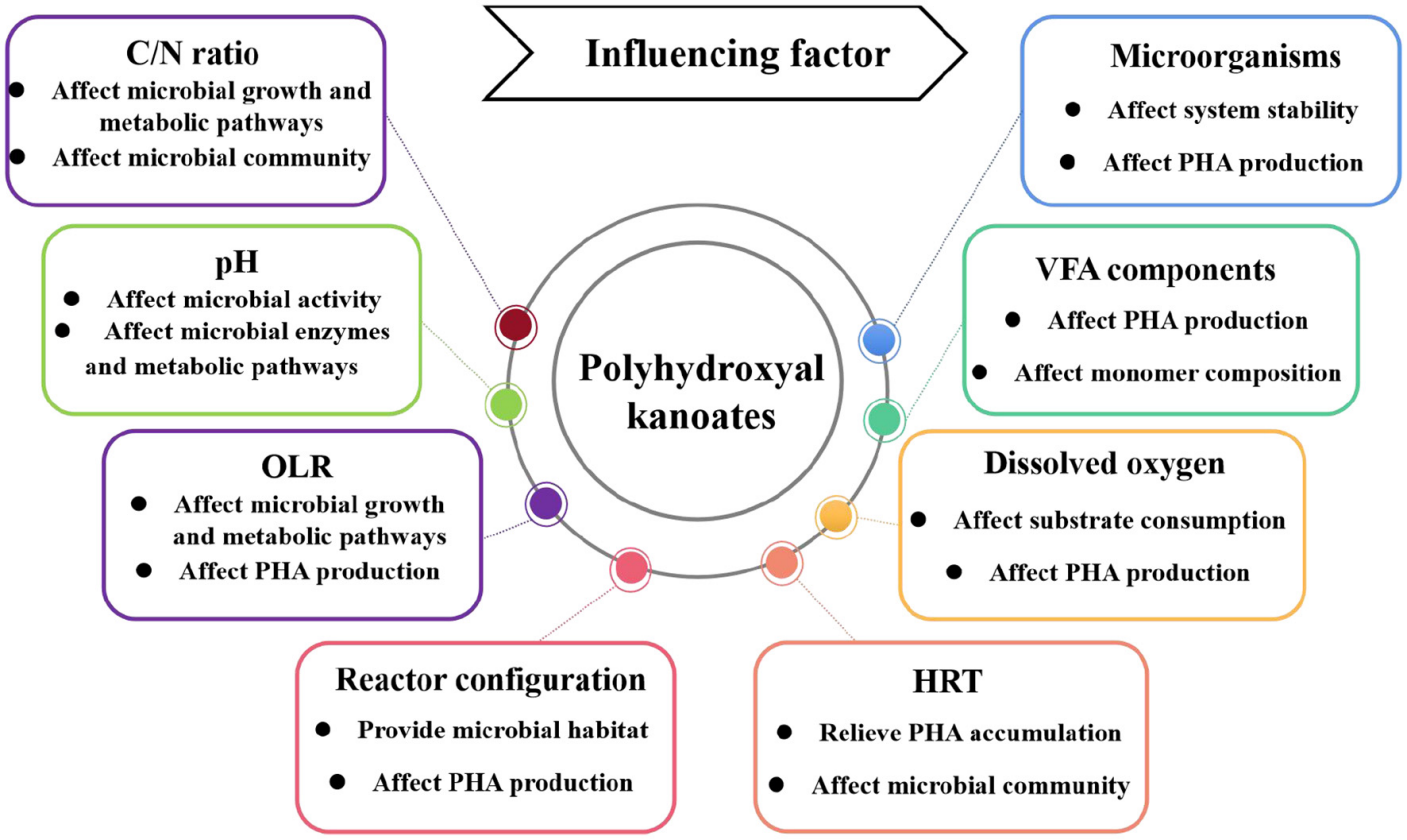
Factors affecting the PHA production from food waste digestate.

PHA generated from food waste digestate is influenced by factors such as VFA components, pH, C/N ratio, dissolved oxygen, OLR, and carbon source composition (Figure 3). These factors can influence microbial community structure, microbial activity, and microbial metabolic pathways, which further affect the PHA production. Optimization of these influencing factors can maximize PHA production, which is essential for industrial-scale applications.

#### 4.3.1 VFA components

VFA components directly influence the PHA production and its monomer composition. Modulation of key organic acid ratios in VFA components is essential for the synthesis of functionally specific PHA (Fu et al., 2023). Generally, even-carbon VFAs (acetate and butyrate) are synthesized as HB, while HV can be synthesized in the presence of propionate, which in turn produces HBV (Chacón et al., 2024). An increase in the proportion of propionate promotes HV production using mixed VFAs as a carbon source, whereas valerate increases HV production by directly generating 3-hydroxy-pentanoyl (Rajesh Banu et al., 2021). Butyrate is more effective in synthesizing PHA than acetate and propionate because butyrate does not require NADH for PHA synthesis (Tao et al., 2022). Microorganisms in the synthesis of PHA from mixed VFAs firstly utilize acetate for their growth, which further increases the storage capacity of PHA in the microorganisms to obtain a higher yield of PHA. Even-carbon VFAs are utilized preferentially, and odd-carbon VFAs are utilized later (Sekoai et al., 2022). This is mainly due to the increased demand for acetyl-CoA during PHA synthesis, which further leads to an increase in the consumption of its precursor, even-carbon VFAs. Thus, the performance and yield of PHA are directly influenced by the ratio of odd-even numbered acids in the VFA fractions. However, VFAs synthesized from organic acids greatly increase the production cost of PHA and limit its large-scale production.

VFAs obtained from anaerobic fermentation of food waste offer the possibility of large-scale PHA production. Chandra et al. (2023) reported that acetate and butyrate from food waste were the main precursors with the highest utilization efficiency, followed by propionate and valerate during PHA synthesis. Raunhan et al. (2023) used VFAs in food waste digestate for PHA generation by *T. mechernichensis*, and acetate and propionate were the main precursors, followed by butyrate. Matos et al. (2021) achieved the highest PHA productivity and content using VFAs rich in butyrate obtained from fruit waste by mixed microbial culture in a pilot plant. Thus, acetate, propionate, and butyrate obtained from anaerobic fermentation of food waste are more suitable as precursors for PHA generation. Meanwhile, the mass fraction of odd–even acids in VFAs is directionally regulated to optimize PHA properties. Targeted VFA generation by anaerobic fermentation of food waste becomes a critical step for efficient PHA generation. However, the complex food waste composition increases the difficulty of targeted VFA production, and the components of food waste digestate are complex, with a large number of non-VFA substances that will inevitably impact the performance of PHA. For example, Rangel et al. (2023, 2024) found that ethanol significantly inhibited PHA content and yield.

#### 4.3.2 C/N ratio

Carbon and nitrogen are essential nutrients for microbial growth, and the C/N ratio directly affects microbial growth, reproduction, and substance synthesis (Wang et al., 2023). A high C/N ratio favored the acquisition of a community with strong PHA synthesis capacity due to the ability of these bacteria to adapt to the nutrient imbalance conditions. A low C/N ratio increased the biomass of cells and decreased the ability of the mixed community to synthesize PHA due to competition from stray bacteria (Chavan et al., 2023). Raunhan et al. (2023) found that *T. mechernichensis* achieved 23.9% of PHA at an optimal C/N ratio of 20 with food waste digestate as substrate. Sanchez-Valencia et al. (2021) investigated the effect of C/N ratios of the range 13.3–42.1 on PHA yield using municipal solid waste digestate as substrate, finding that a C/N ratio of 23.3 favored PHA synthesis. *P. putida* also achieved a PHA content of 56% at high nutrient (25 mmol ${\text{NH}}_{4}^{+}$) with a high carboxylate concentration (C/N ratio of 26.5) accumulated from food waste (Chandra et al., 2023). Therefore, high C/N ratios may be more favorable for PHA accumulation from food waste; however, excessively high ratios are not beneficial.

The high PHA content obtained under nitrogen-limited conditions may be attributed to the inhibition of protein synthesis, with PHA becoming the major product (Wen et al., 2010). Additionally, the changes in the C/N ratio can significantly affect the PHA composition. Silva et al. (2017) reported that when the C/N ratio increased from 14.3 to 17.9, the percentage of PHV in PHA decreased from 20% to 12%. The changes in the C/N ratio of the substrate affect the yield and monomer composition of PHA during PHA generation. Thus, maintaining a stable C/N ratio is an effective strategy for efficient targeted PHA generation.

#### 4.3.3 pH

pH is one of the most important parameters in PHA synthesis. A pH level that is too high or too low pH can influence the enzymes and metabolic pathways of microorganisms, thereby reducing microbial activity (Catherine et al., 2022). Besides, pH affects the structure of the complex microbial community, which further alters the amount and type of PHA. The majority of studies have shown that microorganisms maximize PHA content at neutral pH conditions (Liang et al., 2024; Liu S. et al., 2024). In contrast, Villano et al. (2010) found that PHA yield increased as pH increased from 7.5 to 9.5, and the proportion of PHV increased accordingly. In the case of food waste digestate as a substrate, Dan et al. (2023) found that neutral or slightly alkaline (pH 7.0–8.0) environments favored PHA synthesis, while acidic pH was not conducive to PHA synthesis. Therefore, a neutral pH is more favorable for PHA synthesis using food waste digestate as substrate.

#### 4.3.4 OLR

OLR affects microbial growth and its metabolic pathways, which further affect PHA synthesis. Typically, an appropriate increase in OLR can increase biomass growth, further improving PHA production, while too high OLR may lead to inhibitory effects or microbial imbalances, which further affect the PHA yield. Fang et al. (2019) found that a moderate OLR of 2.4 g chemical oxygen demand (COD)/L·day favored PHA generation using rice winery wastewater as substrate, with a PHA yield of 0.23 g/g. de Oliveira et al. (2019) explored the increase in organic loading from 1.0 to 7.1 g COD/L·day on PHA yield using sugarcane stillage as substrate, and observed that the highest PHA yield (0.60 g/g) was obtained when the OLR was 4.5 g COD/L·day. Matos et al. (2021) achieved the highest PHA productivity and content (8.1 g PHA/L·day and 80.5%) with a high OLR (8.7 g COD/L·day) by mixed microbial culture at a pilot plant using fruit waste as substrate. Thus, appropriately increasing the OLR can increase PHA production according to the actual situation of the process. However, there is no consensus on the OLR for PHA generation from food waste, and further research is still needed.

### 4.4 Recent advances in PHA production from food waste digestate

#### 4.4.1 Pure microbial culture

Table 2 shows that many bacteria utilize VFAs from food waste as a carbon source for PHA generation, and the VFAs composition determines the yield and type of PHA. In many industrial processes, pure bacterial culture was utilized for PHA production. *R. palustris* used VFAs generated from food waste to produce PHA, reaching 49% of content, and VFAs with even numbers of carbon were synthesized as 3HB, and VFAs with odd numbers of carbon were synthesized as 3HV (Dan et al., 2023). *P. putida* achieved a PHA content of 56% at nutrient control with a high carboxylate concentration accumulated from food waste, and acetate, propionate, and butyrate were the main precursors for PHA synthesis (Chandra et al., 2023). Noted that *C. necator*, as an efficient producer of PHA, reached 56–87% utilizing food waste as a carbon source (Hafuka et al., 2011; Vu et al., 2022).

**Table 2:** Food waste-oriented VFAs for PHA generation using pure bacterial culture or mixed microbial culture.

**Substrate**	**VFA composition**	**Culture**	**Reactor type**	**PHA content (%)**	**PHA yield (g/g VFAs)**	**PHA production rate (g/L/h)**	**PHA type**	**References**
Food waste	Acetate, propionate, butyrate, hexanoate	*Cupriavidus necator*	Batch	56.0	0.63	0.05	3HB-co-3-HV	Vu et al., 2022
Food waste	Acetate, propionate, butyrate, isovalerate	*C. necator*	Batch	77.5	0.27	0.04	3HB	Khatami et al., 2022
Food waste	Acetate, propionate, butyrate, isovalerate	*Burkholderia cepacia*	Batch	54.9	0.17	0.02	3HB	Khatami et al., 2022
Food waste	Acetate, propionate, butyrate	*Rhodopseudomonas palustris*	Batch	48.6	nd	nd	3HB, 3HV	Dan et al., 2023
Fruit waste	Acetate, butyrate	*C. necator*	Batch	63.0	0.27	0.06	3HB	Martinez et al., 2016
Food waste	Lactate, acetate, propionate	*C. necator*	Batch	87.0	0.18	0.03	3HB-co-3-HV	Hafuka et al., 2011
Food waste	Acetate, propionate, butyrate	*Halomonas boliviensis*	Batch	70.0	nd	0.34	3HB-co-3-HV	García-Torreiro et al., 2016
Cheese whey	Hexanoate, octanoate	*C. necator*	Batch	71.0	0.60	0.21	3HB	Domingos et al., 2018
Food waste	Acetate, propionate, butyrate, valerate	Mixed culture	Batch	36.9	nd	nd	3HB, 3HV	Reddy and Mohan, 2012
Food waste	Acetate, butyrate, hexanoate	Mixed culture	Batch	43.5	0.08	0.11	3HB, 3HV	Perez-Zabaleta et al., 2021
Cheese whey	Acetate, propionate, butyrate	Mixed culture	Batch	45–50	nd	nd	3HB, 3HV	Lagoa-Costa et al., 2022
Fruit waste	Acetate, butyrate, hexanoate	Mixed culture	Batch	71.3	nd	0.14	HB, HV, HH	Silva et al., 2022
Cheese whey	Acetate, butyrate, hexanoate	Mixed culture	Batch	32.5	0.25	0.07	3HB, 3HV, 3HH	Iglesias-Iglesias et al., 2021
Cheese whey	VFA mixtures	Mixed culture	Batch	43	0.55	0.11	HB, HV	Oliveira et al., 2018
Food waste	VFA mixtures	Mixed culture	Batch	47.9	0.13^a^	0.83	3HB, 3HV	Wu et al., 2022

nd, not detected; ^*a*^The unit of this value is g/g volatile solids.

#### 4.4.2 Mixed microbial culture

Composite bacteria domesticated from activated sludge or directly utilizing the microbial community of activated sludge to synthesize PHA can effectively reduce the PHA generation cost compared to pure culture (Fu et al., 2023). Some studies have attempted to combine VFA fermentation and PHA generation in mixed microbial culture. Amulya et al. (2015) evaluated the PHA generation in a multi-stage operation using food waste as substrate, and PHA content reached 16%−24% accompanied by VFA removal up to 84%−88%. PHA content in an enriched salinophilic mixed microbial culture reached 33% using food waste digestate as substrate (Wang et al., 2018). Lagoa-Costa et al. (2022) used the co-fermentation digestate of cheese whey and beer wastewater as a carbon source for PHA generation in mixed microbial culture, with PHA content reaching 45%−50%, and found that VFA components in the fermentation digestate significantly affected the microbial community composition. In general, mixed microbial cultures have a lower capacity to produce PHA compared to pure culture (Table 2). This might be mainly due to the low biomass density in the mixed microbial cultures (Wang et al., 2021).

#### 4.4.3 Pilot-scale PHA production

Pilot-scale PHA production is a necessary step prior to industrial PHA production. Some studies have explored the pilot-scale PHA production from food waste to achieve commercialization levels. Marreiros et al. (2023) successfully converted lab-scale PHA production using acetate and butyrate obtained from salmon silage as substrates to pilot scale, and PHA content and yield reached 50.4% and 0.189 g/g, respectively, higher than using cheese whey as a substrate in mixed microbial culture (Lagoa-Costa et al., 2022; Oliveira et al., 2018). Kacanski et al. (2023) achieved a pilot-scale PHA production with *Pseudomonas citronellolis* using acetic acid as a carbon source, and a total of 1.75 kg of PHA was produced, corresponding to a PHA yield of 0.051 g/g. In a pilot plant for PHA generation from a mixed microbial culture, utilizing food waste as feedstock for 180 days of operation, the PHA content achieved 48% in a conventional three-stage process (Wu et al., 2022), while the greenhouse gas emission was as high as 70.2 kg CO_2_-eq/kg PHA. Silva et al. (2022) achieved the hydroxyhexanoate (HHx)-containing PHA production in a conventional three-stage process at pilot scale, utilizing fruit waste as feedstock, and PHA content reached 71.3%. Matos et al. (2021) achieved the highest PHA productivity and content (8.1 g PHA/L·days and 80.5%) through a series of regulatory strategies (such as high OLR and continuous feeding) by mixed microbial culture at a pilot plant using fruit waste as a substrate. The above research lays the foundation for the scale-up production of PHA using food waste.

## 5 Techno-economic and environmental analysis for PHA generation

Presently, the cost competitiveness and environmental friendliness are the main factors limiting the industrial PHA generation as a viable alternative to petrochemical plastics. The production cost of PHA using a single substrate (e.g., glycerol, glucose, sucrose) is $3.0–6.1/kg, significantly higher than the current market price of polypropylene and polyethylene terephthalate ($1.1–1.4/kg) (Table 3). To further improve the economic viability of PHA generation, the development of various food wastes as inexpensive carbon sources can effectively reduce production costs. Gnaim et al. (2025) reported that the lowest achievable cost for producing 1,871 ton/year of PHA reached $4.6/kg using *Cobetia amphilecti* from celery waste. On a large scale of 10,000 ton/year, the production cost of PHA was estimated to be $1.5–3.0/kg utilizing sugarcane bagasse as a substrate (Nieder-Heitmann et al., 2019). Meanwhile, in a study with an annual production of 50,000 ton of PHA using rice straw and softwood as substrates, the production cost of PHA was $3.5–3.9/kg, which was lower than that of glucose (Ozturk et al., 2025). Additionally, Zahari et al. (2015) reported that the estimated production cost of oil palm leaves as substrate was $3.4/kg due to the lower production efficiency, slightly higher than that using sugarcane bagasse ($2.1/kg) (Nieder-Heitmann et al., 2019) and crude glycerol ($1.5–2.0/kg) (Thanahiranya et al., 2025). Thus, PHA generation from food waste as a substrate contributes to reducing costs. Meanwhile, further reductions in production costs and improvements in PHA production efficiency are still needed to maintain cost competitiveness with petrochemical peers.

**Table 3:** Techno-economic and environmental analysis of PHA production.

**Types**	**Scale of study**	**PHA production cost ($/kg)**	**Global warming potential (kg CO_2_-eq/kg PHA)**	**Abiotic depletion potential (MJ/kg polymer)**	**References**
Cheese whey	Lab scale	3.0	5.9	79.5	Asunis et al., 2021
Paper mill or food industry wastewater	Lab scale	1.5–2.1	2.2–4.3	106–158	Fernández-Dacosta et al., 2015
Soybean oil	Theoretical simulations	nd	4.4	50.3	Kookos et al., 2019
Sunflower meal and crude glycerol	Theoretical simulations	12.5	0.6	61.7	Kachrimanidou et al., 2021
Glycerol	Lab scale	5.9–6.1	0.6	nd	Leong et al., 2017
Sugarcane bagasse	Theoretical simulations	2.1	4.2	nd	Nieder-Heitmann et al., 2019
Food waste	Pilot scale	nd	70.2	nd	Wu et al., 2022
Crude glycerol	Lab scale	1.5–2.0	5.4–8.2	nd	Thanahiranya et al., 2025
Polypropylene	Industrial scale	1.1	1.6–2.2	70.2–71.3	Mannheim and Simenfalvi, 2020
Polyethylene terephthalate	Industrial scale	1.4	2.2	69.0	Nieder-Heitmann et al., 2019

nd, not detected.

Life-cycle assessment (LCA) can evaluate the environmental impact of raw material selection on the PHA production process. Kookos et al. (2019) estimated the greenhouse gas emissions of PHA generation from soybean oil, reaching 4.4 kg CO_2_-eq/kg PHA, which was higher than the study of Akiyama et al. (2003), who used the same carbon source, ranging from −0.2 to 0.8 kg CO_2_-eq/kg PHA. The latter study attributed the low CO_2_ emissions to absorption of CO_2_ by soybean plants from the air, which was advantageous compared to other food waste, such as cheese whey (5.9 kg CO_2_-eq/kg PHA) (Asunis et al., 2021) and sugarcane bagasse (4.2 kg CO_2_-eq/kg PHA) (Nieder-Heitmann et al., 2019). Wu et al. (2023) first reported that a pilot-scale PHA generation utilizing food waste as the feedstock achieved a PHA content of 47.9%, and the greenhouse gas emission was 70.2 kg CO_2_-eq/kg PHA, which was more than 5 times that of the other references. Also, the fossil consumption potential was about 10 times that of petroleum-based polyethylene plastics. As a whole, there is no environmental advantage in terms of carbon footprint of PHA production from food waste compared to conventional plastics (1.6–2.2 kgCO_2_-eq/kg PHA) (Lim et al., 2023). The higher global warming potential might be due to the low PHA yield from food waste fermentation and the complex PHA extraction process (Khatun and Wright, 2025; Ramos et al., 2019). Thus, the economic and environmental impacts of PHA generation from food waste need to be further studied, and ongoing research should aim to increase PHA yield and quality and optimize the PHA extraction process, which further improve the economic and environmental viability of PHA on a commercial scale.

In conclusion, the techno-economic and environmental analysis of PHA production from food waste has great differences due to the differences in substrates and scales, which impose substantial limitations on comparing them. This section mainly focuses on the comparison of trends and qualitative insights rather than on direct numerical comparisons.

## 6 Challenges and prospects

**Figure 4:**
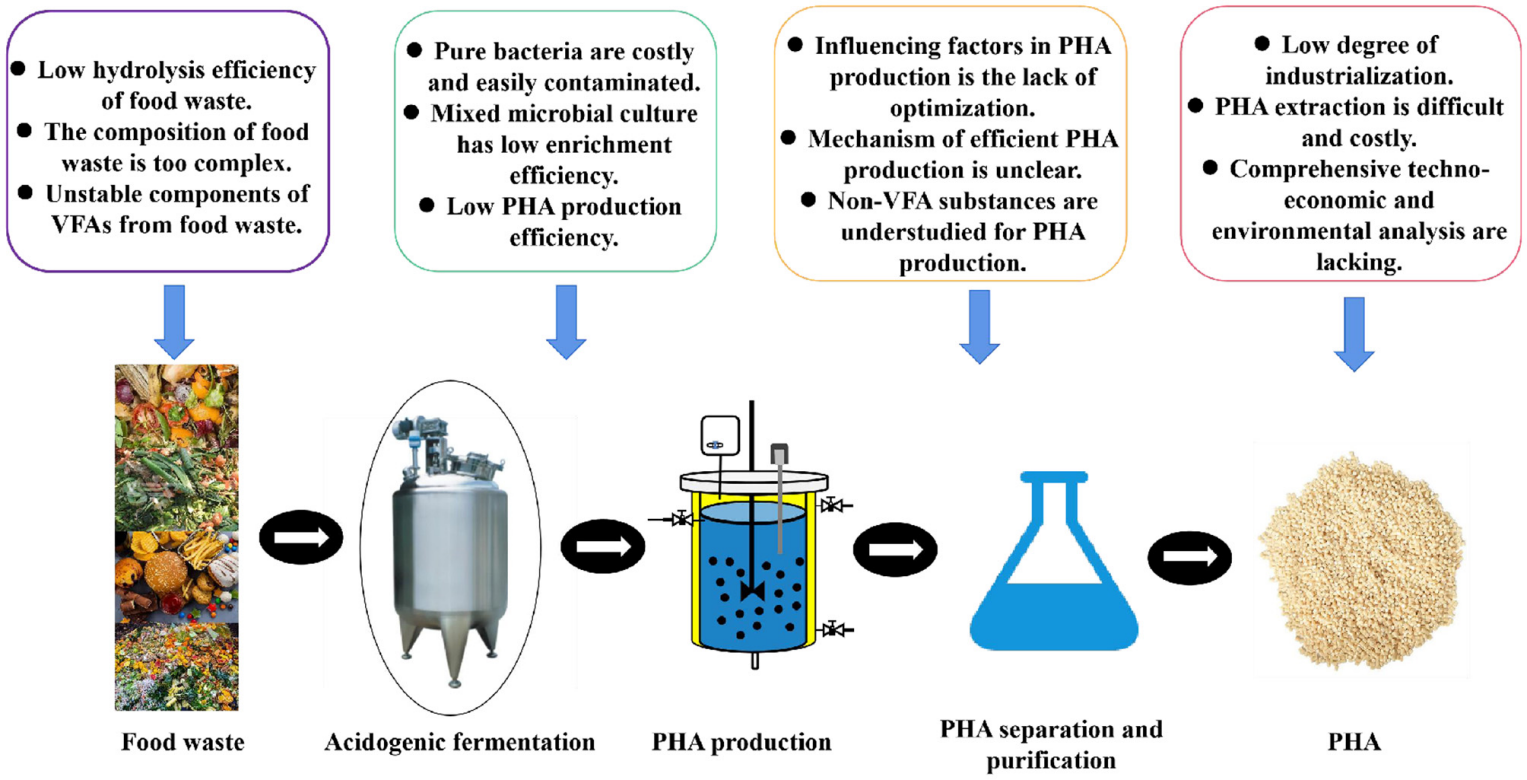
Challenges in food waste-VFAs-PHA generation.

Though VFAs obtained from food waste to generate PHA greatly reduce costs, providing a new model for PHA production (Liang et al., 2024), some challenges still need to be addressed (Figure 4).

(1) VFA components obtained from anaerobic fermentation of food waste determine the content of PHA and its monomer composition. However, the inherent properties of food waste (such as high salinity, variability in composition, and nutrient imbalance) significantly influence the stability and reproducibility of VFA production, which in turn affects PHA production. PHA synthesis typically employs a “two-stage fermentation,” involving rapid microbial growth followed by stimulation of substantial PHA accumulation under nutrient-limited conditions. This process demands extremely precise control of dissolved oxygen, pH, temperature, and feeding strategies. However, the unstable fractions of food waste digestate significantly complicate these control requirements. Thus, stable VFA fractions obtained from food waste fermentation are crucial for achieving efficient PHA production.

(2) Due to the complex composition of food waste digestate, the majority of the known PHA-producing strains or mixed communities face low production efficiency and are susceptible to bacterial contamination, making it difficult to meet the requirements of industrial production. More excellent strains or mixed communities through natural screening, mutation breeding, or genetic engineering still need to be developed. Meanwhile, the resources for strains or mixed communities resistant to extreme environments (e.g., oils, salts, acids, and alkalis) are being further developed, thereby increasing microbial resistance and reducing the risk of bacterial contamination. In addition, the majority of the research studies about PHA generation utilizing food waste digestate focused on the lab level, and a few research studies explored it on a pilot scale. Further pilot-scale research studies need to be verified.

(3) Currently, most research on PHA production from food waste digestate focuses on efficiency, often neglecting the quality and monomer composition of PHA. The appropriate strategies for increasing the natural copolymerization of PHA within microorganisms need to be further explored to increase the PHA quality. Some new monomers, structures, and processing techniques for PHA performance still need to be developed, so that PHA properties can approach or even surpass those of petroleum-based plastics. Currently, PHA extraction technologies are costly, highly toxic, and environmentally unfriendly. Green, efficient, and economical PHA extraction technologies require further development. Finally, a comprehensive techno-economic and environmental analysis of PHA generation utilizing VFAs from food waste is required to assess the feasibility of PHA as an alternative to petrochemical plastics.

## 7 Conclusion

VFAs derived from food waste as carbon sources hold promise for large-scale and commercialized production of low-cost PHA. VFA components directly affect PHA production, its monomer composition, and performance. Even-carbon VFAs are synthesized as HB, and odd-carbon VFAs are synthesized as HV. Butyrate synthesized PHA more efficiently than acetate and propionate. Pretreatment of food waste and the targeted regulation of anaerobic fermentation systems to produce VFAs are beneficial for PHA generation. The pH, C/N ratio, and OLR, etc., are the main factors affecting PHA generation. Also, mixed microbial culture and genetic engineering improve PHA synthesis ability, which is beneficial for further optimization and scale-up purposes. In addition, techno-economic and environmental analysis for PHA generation using VFAs obtained from food waste requires further comprehensive evaluation.
